# Mucin Promotes Rapid Surface Motility in *Pseudomonas aeruginosa*

**DOI:** 10.1128/mBio.00073-12

**Published:** 2012-05-01

**Authors:** Amy T. Y. Yeung, Alicia Parayno, Robert E. W. Hancock

**Affiliations:** Centre for Microbial Diseases and Immunity Research, University of British Columbia, Vancouver, British Columbia, Canada

## Abstract

An important environmental factor that determines the mode of motility adopted by *Pseudomonas aeruginosa* is the viscosity of the medium, often provided by adjusting agar concentrations *in vitro*. However, the viscous gel-like property of the mucus layer that overlays epithelial surfaces is largely due to the glycoprotein mucin. *P. aeruginosa* is known to swim within 0.3% (wt/vol) agar and swarm on the surface at 0.5% (wt/vol) agar with amino acids as a weak nitrogen source. When physiological concentrations or as little as 0.05% (wt/vol) mucin was added to the swimming agar, in addition to swimming, *P. aeruginosa* was observed to undergo highly accelerated motility on the surface of the agar. The surface motility colonies in the presence of mucin appeared to be circular, with a bright green center surrounded by a thicker white edge. While intact flagella were required for the surface motility in the presence of mucin, type IV pili and rhamnolipid production were not. Replacement of mucin with other wetting agents indicated that the lubricant properties of mucin might contribute to the surface motility. Based on studies with mutants, the quorum-sensing systems (*las* and *rhl*) and the orphan autoinducer receptor QscR played important roles in this form of surface motility. Transcriptional analysis of cells taken from the motility zone revealed the upregulation of genes involved in virulence and resistance. Based on these results, we suggest that mucin may be promoting a new or highly modified form of surface motility, which we propose should be termed “surfing.”

## IMPORTANCE

An important factor that dictates the mode of motility adopted by *P. aeruginosa* is the viscosity of the medium, often provided by adjusting agar concentrations *in vitro*. However, the gel-like properties of the mucous layers that overlay epithelial surfaces, such as those of the lung, a major site of *Pseudomonas* infection, are contributed mostly by the production of the glycoprotein mucin. In this study, we added mucin to swimming media and found that it promoted the ability of *P. aeruginosa* to exhibit rapid surface motility. These motility colonies appeared in a circular form, with a bright green center surrounded by a thicker white edge. Interestingly, bacterial cells at the thick edge appeared piled up and lacked flagella, while cells at the motility center had flagella. Our data from various genetic and phenotypic studies suggest that mucin may be promoting a modified form of swarming or a novel form of surface motility in *P. aeruginosa*.

## Introduction

*Pseudomonas aeruginosa* is a Gram-negative opportunistic pathogen that can be found free-living in water and soil and also causes infections in a variety of animals and plants (1). Notably, it is commonly associated with nosocomial infections, particularly lung infections, and is the dominant pathogen in chronic cystic fibrosis (CF) pulmonary infections, persisting in the lungs and inducing serious inflammation that destroys healthy host tissue (2, 3). *P. aeruginosa* infections are particularly difficult to treat due to the bacterium’s intrinsic resistance to a broad spectrum of antimicrobial agents and its repertoire of virulence factors (4).

Motility plays an important role in the pathogenesis of *P. aeruginosa* (5, 6) and is crucial to the ability of *P. aeruginosa* to colonize the host and form biofilms (7). *P. aeruginosa* is known to exhibit three major forms of motility: (i) flagellum-mediated swimming in an aqueous environment and at low agar concentrations (<0.3% [wt/vol]), (ii) type IV pilus-mediated twitching on solid surfaces (1% [wt/vol] agar) or at the interstitial surface between the agar and plastic or glass (8, 9), and (iii) swarming on semisolid (viscous) surfaces (0.5 to 0.7% [wt/vol] agar), with amino acids serving as the nitrogen source (10). Swarming is a social phenomenon that involves rapid coordinated movement of bacteria across a semisolid surface, often typified by dendritic (strain PA14)- or solar flare (strain PAO1)-like colonial appearances. Several studies have shown that *P. aeruginosa* requires its flagella and type IV pili to swarm (10–12), and swarmer cells have two polar flagella and are elongated compared to the normally singly flagellated and shorter swimming cells (10). Biosurfactants produced by the bacteria, such as rhamnolipids and 3-(3-hydroxyalkanoyloxy) alkanoic acids (HAAs), are involved in swarming motility, as they aid in overcoming the surface tension between the bacterial cells and their environment (10, 13). The bacterium’s quorum-sensing (QS) systems, *las* and *rhl*, also play a role in swarming, possibly by regulating production of rhamnolipids and HAAs (10, 14). In addition to physical changes, swarmer cells overexpress hundreds of genes, including most virulence-related genes, and exhibit elevated adaptive resistance to a variety of antibiotics (11), while more than 230 gene products, including 35 regulators, are required for swarming (12), indicating that swarming is a complex adaptation/lifestyle change rather than just a form of motility.

Besides swimming, twitching, and swarming, in the absence of both flagella and type IV pili, *P. aeruginosa* has recently been shown to exhibit sliding/spreading motility on semisolid surfaces (15). Murray and Kazmierczak demonstrated that sliding motility requires rhamnolipid production and responds to many of the same regulatory proteins and environmental cues as swarming motility but is actually inhibited by the presence of pili (15).

Due to the difficult nature of studying motility in a living host, previous *in vitro* motility studies have suggested swarming as a likely mode of motility utilized by *P. aeruginosa* to colonize the lung based on the conditions that promote swarming motility *in vitro* (semisolid agar), which mimic the viscous environment of the lung, especially in the case of chronic (mucoid) infections in CF patients, where the lung environment is characterized by the production of copious amounts of mucous. An obvious problem, however, is that under *in vivo* conditions, agar is absent; instead, the gel-like properties of the mucous layer are contributed mostly by the production of mucin.

Mucin is a major component of the respiratory mucus. It is a glycoprotein secreted by the mucosal and submucosal glands. The mucin molecule consists of a polypeptide core with branched oligosaccharide side chains, each of which contains 8 to 10 sugars (16). Molecular cross-linking of this structure contributes to the viscoelastic property of mucus (17).

In this study, we examined the motility of *P. aeruginosa* under conditions that mimic *in vitro*, as closely as possible, the conditions in the CF lung. Motility assay media contained synthetic CF sputum medium (SCFM), developed by Palmer et al. to mimic the nutritional composition of the CF sputum (18), without added NH_4_Cl but with added mucin and DNA. When mucin was added to SCFM swimming agar, at concentrations as low as 0.05% (wt/vol), *P. aeruginosa* was observed to undergo accelerated motility on the surface of the agar. In the presence of mucin, the surface motility colonies of both *P. aeruginosa* strains, PA14 and PAO1, appeared circular, with bright green centers surrounded by thicker white edges. We found that this form of motility was dependent on the presence of an intact flagellum but not type IV pili. While quorum sensing (QS) is important, QS-regulated production of rhamnolipids by *P. aeruginosa* was not required for this form of surface propagation. Microscopic analysis of cells taken from the motility edge revealed that cells were piled up, with the majority of bacterial cells lacking flagella. In contrast, bacterial cells at the center of the motility zone had flagella. Overall, our genetic and phenotypic data led us to suggest that mucin might be promoting a highly modified form of swarming or a new form of surface motility.

## RESULTS

### Mucin promoted the same surface motility pattern for strains PA14 and PAO1.

**FIG 1:**
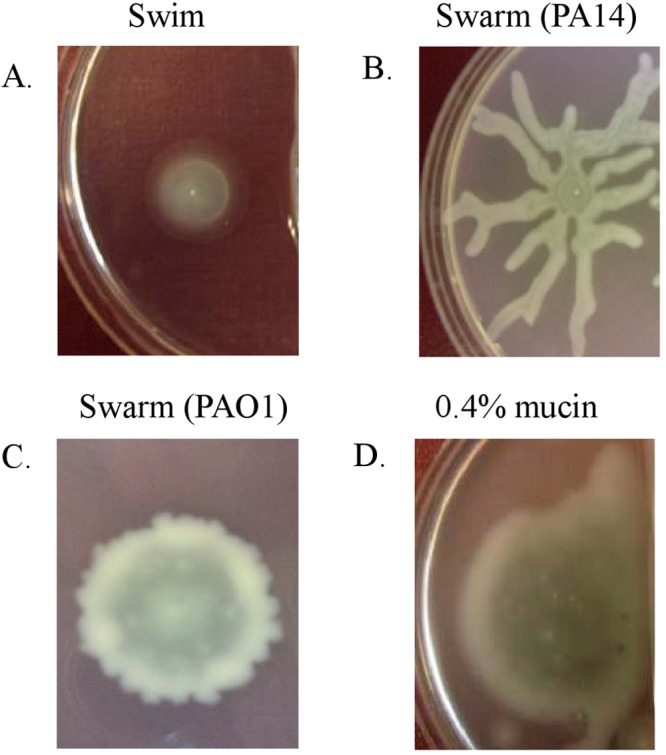
Swimming (A), swarming (B and C), and mucin-promoted (D) motilities of *P. aeruginosa*. Motilities were examined on plates containing 0.3% (wt/vol) agar (swim), 0.5% (wt/vol) agar (swarm), or 0.3% (wt/vol) agar with 0.4% (wt/vol) mucin.

In the presence of a low-percentage (0.2% to 0.35% [wt/vol]) agar, *P. aeruginosa* swims in the submerged water-filled spaces of the agar by the use of its single polar flagellum, resulting in the formation of a halo within the agar layer after overnight incubation at 37°C (Fig. 1A). Interestingly, when mucin was added to “swim” agar, in addition to swimming, *P. aeruginosa* was observed to move relatively rapidly across the surface of the agar. The surface motility zones could be observed when mucin was added at concentrations as low as 0.05% (wt/vol), and the diameter of the surface motility zone increased as the concentration of added mucin increased (up to 1% [wt/vol] mucin tested; Fig. 1D shows an example at 0.4% [wt/vol] mucin). Moreover, the addition of mucin to 0.5% (wt/vol) agar (0.5% agar normally promotes swarming motility of *P. aeruginosa*) changed the surface motility pattern from dendritic to circular, although the diameter of the motility colony remained similar (data not shown). The same surface motility patterns were observed when mucin was spread onto an agar slab. In the presence of mucin, the surface motility colonies of both *P. aeruginosa* strains PA14 and PAO1 appeared circular, with a green center surrounded by a thick white edge. This motility pattern somewhat resembles the solar flare-like colonial swarming pattern of strain PAO1 (Fig. 1C) but differs from the dendritic swarming colony of strain PA14 (Fig. 1B). To better mimic the nutritional composition of the CF sputum, we replaced the typical BM2 (62 mM potassium phosphate buffer [pH 7], 0.1% [wt/vol] Casamino Acids [CAA], 2 mM MgSO_4_, 10 µM FeSO_4_, 0.4% [wt/vol] glucose)-minimal medium in the swim plates with a modified version of the synthetic CF sputum medium (MSCFM) (SCFM [18] without NH_4_Cl) in which NH_4_Cl was excluded. When mucin was added to MSCFM, virtually identical surface motility colonies were observed (data not shown). Moreover, the same motility phenotype was observed when physiological amounts of DNA (1.4 mg/ml) were added to the mucin-MSCFM plates (data not shown). While we used the same DNA concentration (1.4 mg/ml) as Fung et al. used in their synthetic CF sputum growth medium (19), Sriramulu et al. had used a higher concentration of DNA (4 mg/ml) in their artificial CF sputum medium (20). However, in our hands the addition of DNA at ≥4 mg/ml inhibited the growth of *P. aeruginosa* in mucin-MSCFM (Fig. S1). Furthermore, when the surface motilities of *P. aeruginosa* were compared on mucin-containing MSCFM plates (without NH_4_Cl) and mucin-containing SCFM plates (with NH_4_Cl provided at 2.3 mM [18]), no differences were observed in the motility colony morphology, rate of motility zone expansion, or growth of the bacteria.

### Mucin promoted rapid surface motility.

**FIG 2:**
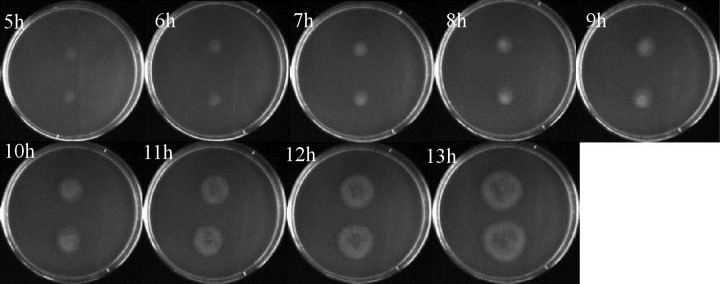
Progression of *P. aeruginosa* PA14 surface motility zones over time. Mid-logarithmic-phase cultures of PA14 WT were spotted onto plates comprised of MSCFM with 0.3% (wt/vol) agar and 0.4% (wt/vol) mucin. Plates were incubated at 37°C, and pictures were taken every hour from h 5 to 13.

When *P. aeruginosa* strain PA14 was spotted onto MSCFM swim plates with mucin (0.05% to 1% [wt/vol]) and incubated overnight at 37°C, the resultant surface motility zones were always greater, in diameter, than the swimming zones observed in the same plate. This led us to examine the rate of this form of surface colonization at various concentrations of mucin. Table 1 shows the average diameters of the motility zones at various concentrations of mucin, while Fig. 2 shows example images of the expanding surface motility zones in the presence of 0.4% (wt/vol) mucin taken every hour from 5 to 13 h. The resultant average motility zone diameter for swimming was 14 mm whereas the swarming zone was 30.4 mm, and the mucin-promoted surface motility zone ranged in size from 21.5 to 38.1 mm in 0.1% to 0.8% (wt/vol) mucin after 13 h of incubation at 37°C. Although swimming motility was found here to be the slowest of the three forms of motility, it should be noted that swimming is more tightly coupled to chemoattractant gradients and thus to growth and that chemoattractant metabolism would limit the rate of motility observed on 0.3% agar plates. Table 1 also shows the average changes in diameter of the motility zones after each hour. For all three forms of motility, the rate of motility increased as time progressed. The rates of swimming motility between h 5 and 13 increased from 0.5 mm/h to 1.5 mm/h, and swarming rates increased from 0 mm/h to 6.4 mm/h. At 0.1%, 0.4%, 0.6%, and 0.8% (wt/vol) mucin, surface motility zones increased from 0.5 to 3.5 mm/h, 1.1 to 4.5 mm/h, 1.3 to 5.2 mm/h, and 1.3 to 5.4 mm/h, respectively.

**TABLE 1:** Average diameters and changes in diameter over time of *P. aeruginosa* strain PA14 motilities in 0.3% (wt/vol) agar (Swim), 0.5% (wt/vol) agar (Swarm), and 0.3% (wt/vol) agar with various concentrations of mucin

Time (h)	Avg diam of motility zone (mm ± SD) or avg rate of change in diam of motility zone (mm/h)*^a^*					
	Swim	Swarm^*b*^	0.1% mucin	0.4% mucin	0.6% mucin	0.8% mucin
	Avg diam of motility zone					
5	4.0 ± 0.1	4.0 ± 0.1	7.0 ± 0.2	8.0 ± 0.2	8.5 ± 0.2	11.0 ± 0.2
6	4.5 ± 0.1	4.0 ± 0.1	7.5 ± 0.1	9.1 ± 0.2	9.8 ± 0.1	12.3 ± 0.1
7	5.5 ± 0.1	4.0 ± 0.1	8.0 ± 0.1	10.5 ± 0.1	11.5 ± 0.1	14.0 ± 0.1
8	6.5 ± 0.2	6.0 ± 0.2	9.0 ± 0.2	12.5 ± 0.2	13.9 ± 0.2	16.5 ± 0.1
9	8.0 ± 0.2	9.0 ± 0.1	11.0 ± 0.2	14.9 ± 0.1	17.0 ± 0.2	19.3 ± 0.2
10	9.5 ± 0.2	13.0 ± 0.2	13.0 ± 0.1	18.0 ± 0.2	20.5 ± 0.1	23.3 ± 0.2
11	11.0 ± 0.3	18.0 ± 0.2	15.0 ± 0.1	22.2 ± 0.1	25.0 ± 0.2	27.9 ± 0.2
12	12.5 ± 0.2	24.0 ± 0.1	18.0 ± 0.2	26.5 ± 0.1	29.8 ± 0.1	32.7 ± 0.3
13	14.0 ± 0.3	30.4 ± 0.2	21.5 ± 0.1	31.0 ± 0.1	35.0 ± 0.2	38.1 ± 0.3
	Avg rate of change in diam of motility zone					
5–6	0.5 ± 0.1	0.0 ± 0.1	0.5 ± 0.2	1.1 ± 0.3	1.3 ± 0.2	1.3 ± 0.2
6–7	1.0 ± 0.1	0.0 ± 0.1	0.5 ± 0.1	1.4 ± 0.2	1.7 ± 0.1	1.7 ± 0.1
7–8	1.0 ± 0.2	2.0 ± 0.1	1.0 ± 0.2	2.0 ± 0.2	2.4 ± 0.2	2.5 ± 0.1
8–9	1.5 ± 0.3	3.0 ± 0.2	1.0 ± 0.3	2.4 ± 0.2	3.1 ± 0.3	2.8 ± 0.2
9–10	1.5 ± 0.3	4.0 ± 0.2	2.0 ± 0.2	3.1 ± 0.2	3.5 ± 0.2	4.0 ± 0.3
10–11	1.5 ± 0.4	5.0 ± 0.3	2.0 ± 0.1	4.2 ± 0.2	4.5 ± 0.2	4.6 ± 0.3
11–12	1.5 ± 0.4	6.0 ± 0.2	3.0 ± 0.2	4.3 ± 0.1	4.8 ± 0.2	4.8 ± 0.4
12–13	1.5 ± 0.4	6.4 ± 0.2	3.5 ± 0.2	4.5 ± 0.1	5.2 ± 0.2	5.4 ± 0.5

^a^ Motility zones were recorded every hour from h 5 to 13 during incubation at 37°C. Results are displayed as means ± SD of triplicate procedures and are representative of three independent experiments.

^b^ For swarming, the same two tendrils on either side of the point of inoculation were measured at every time point.

### Increasing the concentration of mucin did not significantly enhance growth of *P*. *aeruginosa*.

As shown in Table 1, as the concentration of mucin added to the plates increased, the surface motility zone increased. Therefore, we investigated whether the increased motility zones were due to enhanced growth of the bacteria in the presence of mucin. *P. aeruginosa* was grown in liquid MSCFM with 0, 0.1, 0.4, 0.6, or 0.8% (wt/vol) mucin and shaking at 37°C, and aliquots were plated every hour for colony counting. Growth appeared the same at all concentrations of mucin tested (Fig. S2).

### Mucin-promoted surface motility was dependent on flagellar expression but did not require type IV pili.

**FIG 3:**
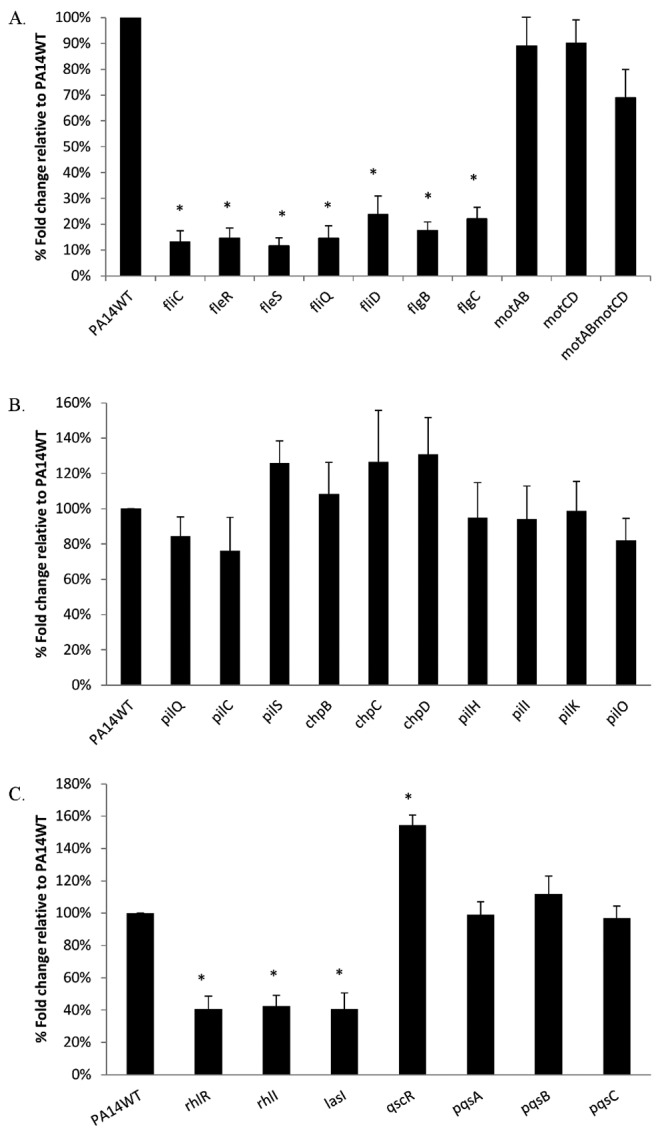
Percent fold changes of surface coverage of PA14 flagellar (A), type IV pilus (B), and quorum-sensing (C) mutants compared to PA14 WT on MSCFM–0.4% mucin–0.3% agar. Motility zones were measured after incubation at 37°C for 13 h. Results shown are means ± standard deviations (SD) for at least three independent experiments, with duplicates for each experiment. Asterisks indicate a statistically significant difference (*P* ≤ 0.05) between the mutants and WT as determined by Student’s *t* test.

**FIG 4:**
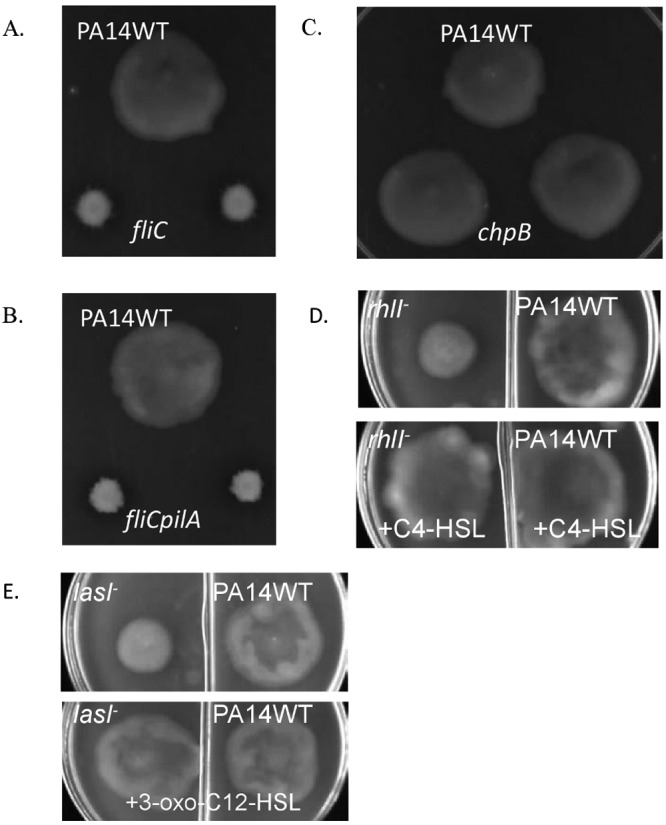
Example images of mucin-promoted surface motilities of PA14 WT and a *fliC* flagellar mutant (A), a *chpB* type IV pilus mutant (B), a *fliC pilA* flagellar and type IV pilus double mutant (C), a *rhlI* quorum-sensing mutant with and without addition of C4-HSL (D), and a *lasI* quorum-sensing mutant with and without addition of 3-oxo-C12-HSL (E). *P. aeruginosa* bacteria were spotted onto MSCFM plates with 0.3% agar and 0.4% mucin and incubated at 37°C for 13 h. For surface motility restoration, 10 µM of either C4-HSL or 3-oxo-C12-HSL was added.

We first examined whether *P. aeruginosa* required its cellular appendages (i.e., flagella, type IV pili) to move on the surface of the mucin plates. As shown in Fig. 3A and 4A, mutants with transposon insertions in genes involved in flagellar biosynthesis, including *fliC*, *fliQ*, *fliD*, *fleR/S*, *flgB*, and *flgC*, were significantly impaired in this form of motility. This suggested the necessity of an intact flagellum for rapid surface motility on mucin plates. *P. aeruginosa* mutants with transposons inserted in genes encoding its stator complex were of particular interest. The stator complex is the stationary element of the bacterial motor, providing energy to turn the flagellum and therefore propel the cell through its environment (21). The stator complex is comprised of four integral membrane proteins, MotAB and MotCD. MotAB and MotCD are to some extent functionally redundant for swimming; therefore, deletion of any given stator does not significantly impair *P. aeruginosa* swimming motility. Nevertheless, while a *motAB* mutant can swarm on 0.5% (wt/vol) agar, a *motCD* mutant is swarming defective at this concentration (21). A *motAB motCD* double mutant is able neither to swim nor swarm. Fig. 3A shows that the PA14 *motAB motCD* mutants exhibited comparable levels of surface motility on the mucin plates compared to the PA14 wild type (WT), while a small reduction in surface motility was observed for the *motAB motCD* quadruple mutant. The results for the PA14 mutants with deletions in *motAB*, *motCD*, and *motAB motCD* were confirmed using the corresponding mutants and complemented strains provided to us by O’Toole (21). Flagellar-driven motility of *P. aeruginosa* was also examined at the center of the mucin-induced motility zone for both the PA14 WT and the *mot* mutants (i.e., *motAB*, *motCD*, *motABCD*). To do this, *P. aeruginosa* was grown on MSCFM plates with 0.4% mucin and 0.3% agar overnight at 37°C. After 13 h, small amounts of cells taken from the edge or center of the motility colony were transferred and taken up into liquid medium, and the cells were immediately observed under the light microscope at ×100 magnification. Observation of *P. aeruginosa* taken from the edge of the mucin-promoted motility colony revealed mostly immotile cells, with a couple of moving cells (data not shown). In contrast, at the center of the mucin-induced motility colony, WT bacteria were highly motile and exhibited typical swimming behavior (running in a relatively straight line occasionally interrupted by tumbling to acquire a new direction). Moreover, all *mot* mutants exhibited motility behavior similar to that of the WT at the center of the motility zone. These data indicated either that flagella rotation was not required for mucin-mediated motility or, more likely, that an alternative to the conventional stator complexes drives flagella rotation in this type of motility. Consistent with the latter explanation, each of the mutants exhibited their published phenotypes in liquid medium (21), with all demonstrating an ability to swim except the quadruple mutant *motABCD*, which was immotile in liquid medium.

Mutants with transposon insertion in genes involved in the assembly of type IV pili exhibited surface motility zones on mucin plates comparable to that of the WT (Fig. 3B and 4B). Moreover, a *fliC pilA* double mutant (defective in flagella and type IV pili [15]) had completely lost its surface motility on mucin plates (Fig. 4C) as expected, given its flagella deficiency.

### Mucin-promoted surface motility was dependent on cell-to-cell signaling.

*P. aeruginosa* possesses three intertwined quorum-sensing systems (*las*, *rhl*, *pqs*) and one orphan autoinducer receptor (*qscR*). Previously, it was demonstrated that in addition to controlling the expression of a number of extracellular virulence factors, the *las* and *rhl* cell-to-cell signaling systems are also required for swarming in *P. aeruginosa* (10). As shown in Fig. 3C, PA14 mutants with transposon insertions in *rhlR*, *rhlI*, and *lasI* were significantly impaired in surface motility in the presence of mucin (no PA14 transposon mutant was available for *lasR*) compared to the PA14 WT. Since *rhlR*, *rhlI*, *lasR*, and *lasI* transposon mutants were available in the PAO1 mutant library (20), these mutants were also tested for their ability to propagate on the surface of mucin plates compared to the PAO1 WT, and we observed defects in the PAO1 mutants similar to those seen with the PA14 mutants (data not shown). The addition of the cognate signals C4-HSL and 3-oxo-C12-HSL restored the surface motility zones in the *rhlI* and *lasI* mutants, respectively, to WT levels, confirming the involvement of quorum sensing in this surface propagation (Fig. 4D and E). Mutations in genes involved in synthesis of *Pseudomonas* quinolone signal (PQS) did not affect this motility. However, a *qscR* transposon mutant exhibited significantly increased surface propagation relative to the PA14 WT. Previous studies showed that the quorum-sensing-control repressor (QscR) transiently represses the expression of several genes activated by LasR or RhlR (22, 23), and this provided a plausible basis for the mutant effects on the surface motility.

Previously, it was demonstrated that *P. aeruginosa* synthesizes rhamnolipids that act as biosurfactants to promote swarming on a semisolid surface. Rhamnolipid production is mainly controlled by the *rhl* system, which regulates the expression of the *rhlAB* operon, encoding rhamnosyltransferase involved in rhamnolipid biosynthesis (10). Here we examined whether rhamnolipid production is required for surface propagation in the presence of mucin. PA14 mutants with transposon insertions in genes involved in the rhamnolipid biosynthesis pathway, including *rhlA* and *rhlB*, exhibited surface motilities in the presence of mucin similar to those seen with the PA14 WT (data not shown). These results were confirmed in the PAO1 mutants with insertions in *rhlA* or *rhlB* genes.

### *P*. *aeruginosa* at the edge of the mucin-promoted surface motility zone lacked flagella.

**FIG 5:**
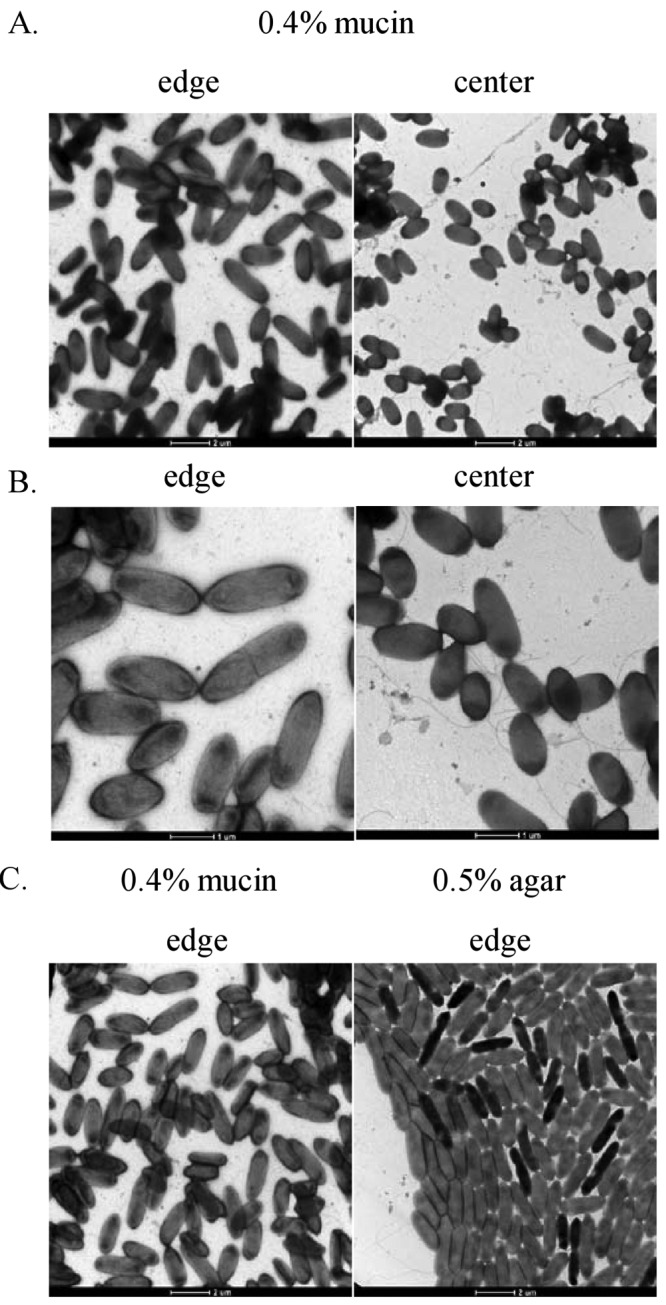
Electron microscopy images of the *P. aeruginosa* strain PA14 WT from motility colonies on 0.5% agar or 0.4% mucin. *P. aeruginosa* bacteria were taken directly from the leading edge and center of the mucin-promoted surface motility zone (A and B) or the leading edge of the swarming motility zone (C). The cells were stained with 1% uranyl acetate and observed using a transmission electron microscope.

We used transmission electron microscopy (TEM) to examine the morphology of *P. aeruginosa* strain PA14 taken from the edge and the center of the mucin-promoted surface motility zone. The method employed involved placing carbon-coated grids directly onto the surface of the motility colonies and then staining the cells with 1% uranyl acetate. The negatively stained cells were visualized using a TEM. As shown in Fig. 5A, the cells on the edge of the mucin-promoted surface motility zone were elongated compared to cells taken from the center of the motility zone. Measurement of the lengths of 10 to 15 random bacteria in each of 4 different squares of a carbon-coated grid placed on the middle or edge of the motility colony revealed that, on average, the length of the bacterial cells from the edge was 1.4- ± 0.2-fold greater than the length of the cells in the middle. Differences in flagellation were also clearly observed: at the edge of the motility zone, the majority of bacteria lacked flagella, with only 9 out of 60 bacterial cells assessed in 4 random grids having flagella, while at the middle, 47 out of 60 cells were flagellated (Fig. 5B). Moreover, while cells taken from swarming tendrils were highly organized (i.e., cells were aligned one next to another in the same direction as the moving tendril), the orientation of bacterial cells taken from the edge of the mucin-promoted surface motility zone was apparently random, with cells overlaying each other (Fig. 5C).

### Promotion of mucin-mediated motility by amino acids and inhibition by ammonium.

**FIG 6:**
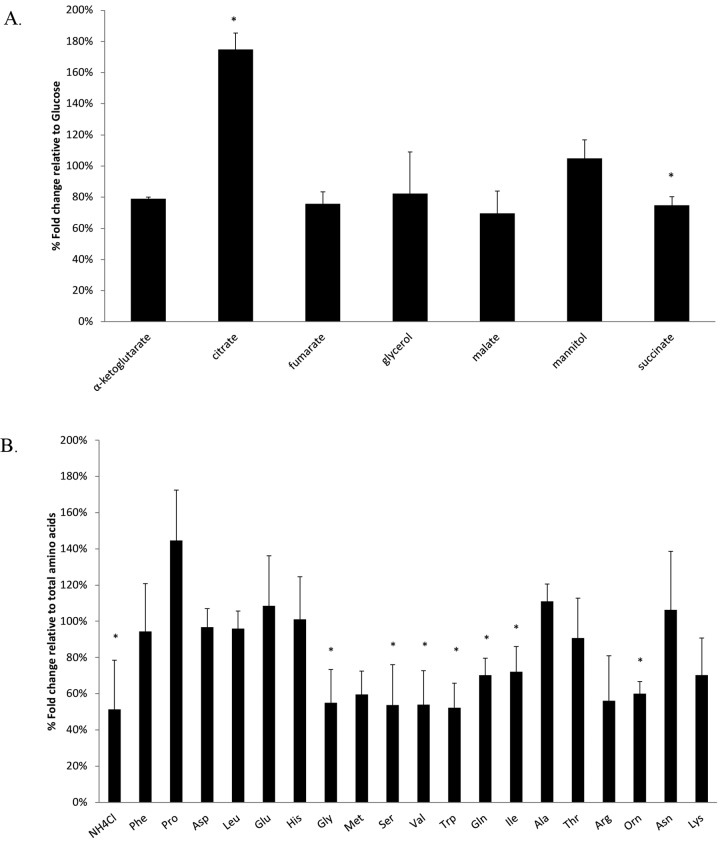
Surface motility of PA14 WT on mucin plates supplemented with various carbon and nitrogen sources. *P. aeruginosa* bacteria were spotted onto MSCFM agar-mucin plates supplemented with (A) glucose (control) or an alternative carbon source or with (B) total amino acids (control) or an alternative nitrogen source and incubated at 37°C for 13 h. Results shown are means ± SD for at least three independent experiments with duplicates for each experiment. Asterisks indicate a statistically significant difference (*P* ≤ 0.05) between the tested condition and control as determined by Student’s *t* test.

**FIG 7:**
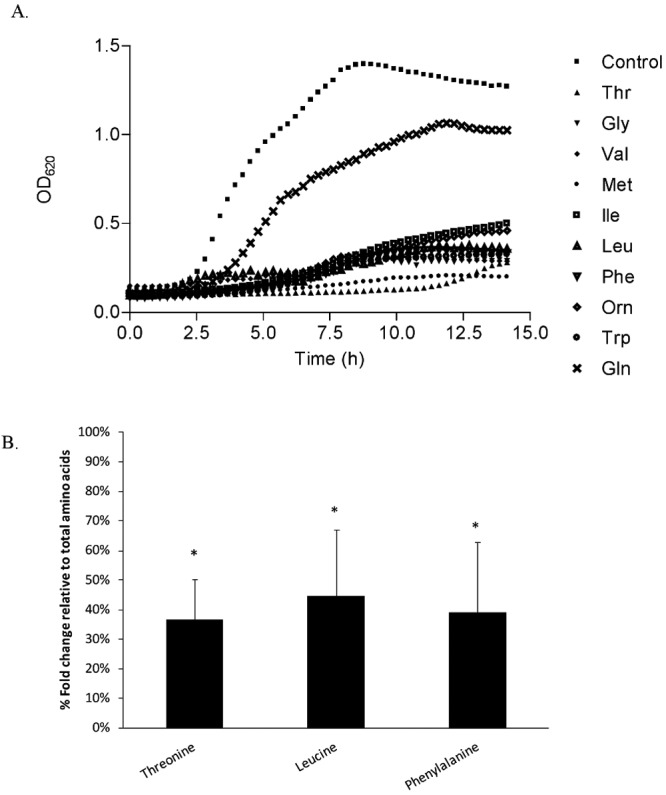
Growth and bacterial cell counts of PA14 WT determined using various amino acids as sole nitrogen (N) sources. (A) *P. aeruginosa* bacteria were grown in liquid MSCFM supplemented with total amino acids (control) or single amino acids serving as the sole N source. Growth was measured at 37°C using a Tecan Spectrofluor Plus reader. (B) *P. aeruginosa* bacteria were spotted onto MSCFM agar-mucin plates supplemented with total amino acids (control) or a single sole N source (as indicated) and incubated at 37°C for 13 h. The entire surface motility colony was resuspended in 1× PBS and serially plated on LB agar plates. Results shown are means ± SD for at least three independent experiments with duplicates for each experiment. Asterisks indicate a statistically significant difference (*P* ≤ 0.05) between the sole nitrogen source and control as determined by Student’s *t* test.

We investigated the effects of different carbon and nitrogen sources on the ability of *P. aeruginosa* to propagate on the mucin plates. To examine the effects of different carbon sources on mucin-promoted motility, we excluded lactate from and replaced glucose in the MSCFM with equimolar amounts of each of the following carbon sources: α-ketoglutarate, succinate, fumarate, citrate, malate, glycerol, and mannitol (Fig. 6A). *P. aeruginosa* exhibited a statistically significant increase in mucin-promoted surface motility when glucose was replaced with citrate and a decrease in surface motility with succinate, even though those carbon sources supported similar growth rates (data not shown), indicating that growth differences could not explain these results. When the total free amino acids in the mucin-MSCFM plates were replaced with an equal concentration (19 mM) of NH_4_Cl as the sole nitrogen source, surface propagation was impaired by about 50% (Fig. 6B); in contrast, this concentration of NH_4_Cl completely eliminated swarming. We also examined the effects of individual amino acids serving as the sole nitrogen sources on the surface motility. The total free amino acids in the normal MSCFM were replaced with equimolar amounts of a single amino acid. When they were provided as the sole nitrogen source, we observed that many of the amino acids tested were able to support this form of surface motility (Fig. 6B). Replacement with glycine, methionine, valine, tryptophan, glutamine, isoleucine, and ornithine gave significantly weaker mucin-stimulated surface motility phenotypes, but the motility zones in these cases still had fold changes greater than 50% compared to the surface coverage of the motility zones in the presence of the total free amino acids in MSCFM. Growth experiments showed that *P. aeruginosa* exhibited moderate growth defects in liquid MSCFM when glutamine was provided as the sole nitrogen source while growth was greatly impaired in the presence of methionine, valine, tryptophan, isoleucine, glycine, or ornithine (Fig. 7A). Although the majority of amino acids that supported strong surface motility exhibited only little to moderate growth impairment (data not shown), leucine, phenylalanine, and threonine each caused significant growth impairments when provided as sole nitrogen sources but supported strong surface motilities (Fig. 6B and 7A). One possibility is that there were fewer bacterial cells in the motility zones with leucine, phenylalanine, or threonine than on normal MSCFM plates. To test this, we resuspended surface motility colonies grown on threonine, leucine, phenylalanine, or normal MSCFM in buffer and performed serial plating. Resultant bacterial cell counts revealed significantly lower numbers of bacterial cells from the surface motility colonies grown on leucine, phenylalanine, or threonine compared to normal MSCFM (Fig. 7B).

### *P*. *aeruginosa* in the mucin-mediated motility zone upregulated the expression of certain genes involved in virulence and resistance.

We used real-time quantitative PCR (RT-qPCR) to examine the expression of selected genes involved in virulence and antibiotic resistance in *P. aeruginosa*, comparing the gene expression of *P. aeruginosa* taken from the edge and center of the surface motility zone with 0.4% mucin to that of bacteria swimming in identical medium without mucin. As shown in Table 2, *P. aeruginosa* in the mucin-mediated motility zone downregulated the expression of the type 3 secretion system (T3SS) (i.e., the fold changes for *pcrV* were −10.2 and −5.9 from the edge and center populations, respectively) and T3SS-secreted factors (i.e., fold changes of *exoT* were −3.5 and −3.3 from the edge and center populations, respectively). In contrast, genes encoding the Xcp type 2 secretion system (T2SS) and its relevant extracellular virulence factors, including elastase, exotoxin A, and lipase, were upregulated compared to those of swimming cells. Genes encoding the biosynthesis of phenazine compounds were also downregulated in both the edge and center populations. Interestingly, while the edge population upregulated genes involved in pyoverdine and pyochelin biosynthesis, the center population downregulated these genes (Table 2).

**TABLE 2:** Fold change in expression of selected genes in *P. aeruginosa* strain PA14 taken from the edge and center of the surface motility zone with 0.4% mucin compared to the results seen with bacteria swimming in identical medium without mucin as determined using RT-qPCR

Gene designation	Name	Fold change (edge)	Fold change (center)
Type III secretion system			
PA0044	*exoT*	−3.5 ± 1.1	−3.3 ± 1.0
PA1706	*pcrV*	−10.2 ± 0.9	−5.9 ± 1.5
PA1708	*popB*	−9.0 ± 1.1	−11.0 ± 1.1
PA1709	*popD*	−6.0 ± 1.4	−3.2 ± 1.1
PA1710	*exsC*	−5.9 ± 1.1	−9.3 ± 1.4
PA1711	*exsE*	−4.2 ± 1.3	−3.3 ± 1.2
PA1713	*exsA*	−6.4 ± 1.1	−8.4 ± 1.1
PA1717	*pscD*	−10.2 ± 1.2	−12.0 ± 3.4
PA1721	*pscH*	−12.3 ± 1.5	−4.9 ± 1.3
PA2191	*exoY*	−6.8 ± 1.9	−3.1 ± 1.0
Type II secretion system			
PA1148	*toxA*	3.1 ± 1.1	5.4 ± 1.8
PA3096	*xcpY*	4.6 ± 1.4	2.2 ± 0.8
PA3104	*xcpP*	7.1 ± 1.3	8.9 ± 2.5
PA3724	*lasB*	72.6 ± 2.5	13.8 ± 3.3
PA4813	*lipC*	5.1 ± 1.5	3.6 ± 1.4
Pyoverdine biosynthesis			
PA2385	*pvdQ*	5.1 ± 1.3	−7.7 ± 2.4
PA2399	*pvdD*	3.3 ± 1.1	−6.2 ± 2.1
PA2426	*pvdS*	6.1 ± 1.6	−5.7 ± 2.1
Pyochelin biosynthesis			
PA4221	*fptA*	4.3 ± 1.5	−4.9 ± 2.3
PA4224	*pchG*	3.6 ± 1.1	−4.5 ± 1.8
PA4226	*pchE*	8.2 ± 1.6	−3.3 ± 1.1
PA4228	*pchD*	4.5 ± 1.3	−4.5 ± 2.1
PA4229	*pchC*	6.6 ± 1.7	−7.9 ± 2.8
Phenazine biosynthesis			
PA1900	*phzB2*	−3.3 ± 1.1	−3.9 ± 1.0
PA4209	*phzM*	−4.6 ± 1.4	−6.8 ± 3.2
PA4210	*phzA1*	−4.9 ± 1.5	−2.9 ± 1.1
PA4211	*phzB1*	−5.4 ± 1.8	−9.0 ± 2.0
PA4213	*phzD1*	−3.9 ± 1.1	−6.9 ± 1.3
PA4216	*phzG1*	−6.3 ± 1.5	−8.4 ± 2.3
Adaptation and resistance			
PA1178	*oprH*	1,173.0 ± 21.5	189.0 ± 14.5
PA1179	*phoP*	181.9 ± 6.8	89.0 ± 19.1
PA1797		4.2 ± 1.9	24.5 ± 6.5
PA3552	*arnB*	188.8 ± 15.7	82.6 ± 33.9
PA4777	*pmrB*	3.2 ± 1.4	24.4 ± 7.9

Moreover, RT-qPCR revealed that genes involved in antibiotic resistance in *P. aeruginosa* (i.e., *pmr* and *arn* operons) were upregulated in *P. aeruginosa* from both the edge and the center of the mucin-promoted surface motility zone. Since the induction of the *pmr* and *arn* operons is known to induce resistance of *P. aeruginosa* to polymyxins and cationic antimicrobial peptides (24), resistance to polymyxin B was tested. Antibiotic discs with polymyxin B (at 9 mg/ml) were placed onto the center of MSCFM plates with 0.3% agar and 0.4% mucin and compared to MSCFM plates with 0.3% agar only (swimming). This was followed by spotting mid-log-phase cultures of *P. aeruginosa* onto these plates and incubating them at 37°C for 13 to 15 h, allowing the motility colonies to grow towards the polymyxin disc. As shown in Fig. S3, the inhibition zones were much larger on the swim plates than on the mucin plates, indicating resistance for the latter.

### Mucin promotion of surface motility may involve lubricant-like action.

**FIG 8:**
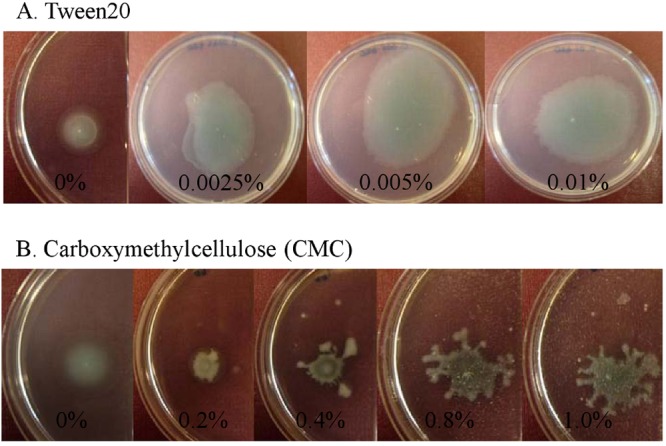
Surface propagation of *P. aeruginosa* strain PA14 WT on wetting or viscosity-enhancing agents. Cultures of *P. aeruginosa* bacteria were spotted onto MSCFM plates with 0.3% (wt/vol) agar and various concentrations of (A) Tween 80 or (B) CMC. Images were taken after incubation at 37°C for 13 h.

As described above, rhamnolipid production was not required for this form of surface motility on mucin plates. This led us to suspect that mucin might be serving as a surrogate for a surfactant to reduce surface tension and promote this form of motility. It has been demonstrated that soluble mucin is both viscous and lubricative (25, 26). For example, the addition of porcine gastric mucin enhances the viscosity and wettability of solutions (27). Basically, the viscosity-enhancing property of mucin allows mucin to bind water, reducing the free water content in the environment and thus increasing the ability of bacteria to slide across the surface. To examine whether the lubricant/wetting properties of mucin affected the surface motility, we replaced mucin with various wetting agents. Replacement of mucin with Tween 20 promoted rapid surface propagation of *P. aeruginosa* (Fig. 8A). The replacement of mucin with Tween 20 did not result in a pattern identical to that of mucin and gave surface motility patterns that were more inconsistent, with variable levels of surface coverage seen in different trials, while the edges of the Tween 20-mediated motility colony were thinner than the rest of the motility colony, in contrast to the consistent surface coverage and thick edges of mucin-mediated motility zones. We also demonstrated that flagellar mutants were able to spread on Tween-containing plates (data not shown). These results were consistent with the suggestion that the improved surface wetness provided by mucin facilitated the ability of bacterial cells to expand on the agar. We also replaced mucin with carboxymethyl cellulose (CMC), which has both lubricative and viscous properties, to examine its effect on surface motility. As shown in Fig. 8B, CMC promoted surface propagation but higher concentrations of CMC were required compared to mucin. At 1% (wt/vol), CMC promoted only about 50% of the surface coverage promoted by 0.4% (wt/vol) mucin. Overall, it was concluded that a unique combination of wetness and viscosity conferred by mucin likely contributed to its effects on surface motility.

## DISCUSSION

The ability of a spectrum of microorganisms, and notably *P. aeruginosa*, to colonize the lungs of CF patients is associated with subsequent lung deterioration and health decline. Motility of these microorganisms has been shown to aid in the initial colonization process (5, 28). The CF lung environment is characterized by the production of copious amounts of mucus (sputum), which covers the surface of epithelial cells. Mucin, being a major component of respiratory secretions, typically at concentrations around 0.5 to 1% (wt/vol) (29–31), gives mucus its gel-like properties and is regarded as an important molecule in the initial colonization by *P. aeruginosa* of the airways of CF patients (32). Due to current limitations in our ability to observe bacterial motility in a host, we constructed an *in vitro* model that mimicked as closely as possible the composition of the CF lung to study motility of *P. aeruginosa* under similar conditions. This *in vitro* motility model utilized the typical carbon, nitrogen, and energy sources for growth of *P. aeruginosa* in a version of SCFM (18) lacking NH_4_Cl, since such a strong N source strongly inhibits swarming (e.g., addition of 1 mM and 2.3 mM NH_4_Cl to SCFM led to around 50% and 70% inhibition of swarming, respectively). Subsequently, we observed that mucin-mediated motility was unchanged when 2.3 mM NH_4_Cl was included in the medium, and even the replacement of all N sources by 19 mM NH_4_Cl inhibited mucin enhanced motility by only 50%. CF sputum also contains DNA at concentrations ranging from 0.6 mg/ml to 36 mg/ml (33–35), possibly reflecting differences in patient disease progression or different methods used to detect DNA. Consequently, different concentrations of DNA have been used in *in vitro* studies designed to mimic the CF lung sputum. Here, DNA was added at 1.4 mg/ml, as suggested by Fung et al. (19), to the mucin-SCFM plates since, at that concentration, DNA did not inhibit growth of *P. aeruginosa*. This concentration also had no effect on mucin-enhanced motility. In contrast, Sriramulu et al. recommended adding DNA at 4 mg/ml (20), but in our hands this DNA concentration completely inhibited the growth of *P. aeruginosa* (Fig. S1). The use of 0.3% agar provided a surface for easy observation of the motility of *P. aeruginosa*, while the water content in the plate mimicked the water content of sputum (36). Under these conditions, the addition of mucin promoted the ability of both *P. aeruginosa* strains, PAO1 and PA14, to exhibit rapid motility across the surface of the agar, resulting in the formation of large green colonies surrounded by thick white edges that resembled the swarming pattern of strain PAO1 but differed from that of strain PA14.

*P. aeruginosa* is well-known for its ability to swim, twitch, swarm, and slide. Swimming is mediated by rotation of the bacterium’s single polar, monotrichous flagellum. Independently of the flagellum, twitching requires type IV pilus filaments that extend the cell body to adhere to a surface and then retract, thus “dragging” the bacterium forward. Swarming motility requires both functional flagella and type IV pili (although certain studies have suggested that swarming does not require functional pili), while sliding motility occurs in the absence of flagella and type IV pili. Mucin-promoted surface motility is independent of type IV pili, suggesting that mucin is not promoting twitching motility. That a *fliC pilA* double mutant is able to slide but unable to propagate on a mucin-containing plate suggested that this form of surface motility is not sliding. Also, the requirement for intact flagellum, but not particular stator flagellar functions, clearly distinguishes this form of surface motility from swimming and conventional swarming motility.

In this study, we have shown that quorum sensing plays an important role in promoting motility on mucin-containing plates, since mutations in the *rhl* and *las* systems significantly impaired mucin-promoted surface motility and motility could be rescued with the cognate homoserine lactones. Although swarming has been shown to be dependent on quorum sensing for the production of the biosurfactant rhamnolipids, swimming and twitching motilities are independent of quorum sensing. Dependence on rhamnolipid production for sliding motility was shown by Murray and Kazmierczak, who demonstrated that a *fliC pilA rhlA* mutant was unable to slide (15). Here we demonstrated that mutations in the genes involved in rhamnolipid biosynthesis, *rhlA* and *rhlB*, did not impair the ability of *P. aeruginosa* to demonstrate rapid surface motility on the mucin plates. It seems likely that rhamnolipid production was not required for this surface motility, because mucin was acting as a surfactant alternative to rhamnolipid or otherwise promoting surfactant activity.

In addition to flagella, type IV pili, and rhamnolipids, classical swarming motility is also dependent on specific carbon-nitrogen sources. Köhler et al. showed that while glucose promoted swarming when provided as the sole carbon source, glycerol and succinate did not (10). Also, swarming motility was shown to be completely inhibited when NH_4_Cl was provided as the sole nitrogen source whereas aspartate, glutamate, and histidine promoted strong swarming phenotypes (10). Carbon and nitrogen source substitutions in the mucin-containing plates revealed that this form of surface motility was less nutritionally restricted than swarming, since it was demonstrated here that this form of mucin-mediated surface motility could occur with a wide variety of carbon and nitrogen sources. Specifically, amino acids, including arginine, aspartate, and leucine, promoted strong surface motility on mucin plates, while these individual amino acids completely abolished swarming. Similarly, NH_4_Cl is unable to serve as the sole nitrogen source to promote swarming motility, and the addition of NH_4_Cl at concentrations as low as 1 mM inhibited swarming. In contrast, the addition of up to 5 mM NH_4_Cl to mucin-MSCFM plates did not affect surface motility, indicating the relative insensitivity of mucin-mediated motility to the type of N source compared to the results seen with swarming.

Examination of *P. aeruginosa* cells from the edge of the mucin motility zones revealed a lack of flagella for the majority of the cells analyzed, although a few bacterial cells still had flagella attached. This is in contrast to *P. aeruginosa* at the edge of a swarm zone, where the majority of bacterial cells possess two polar flagella (10). Moreover, while swarmer cells are highly organized (i.e., cells are aligned one next to another in the same direction as the moving tendril), the orientation of the bacterial cells on the edge of the mucin motility zones appeared random, with bacterial cells overlaying each other. In myxobacteria, the ordering of swarmer cells results from the ability of swarming bacteria to undergo periodic reversals of moving direction in dense populations (37). Therefore, in this study, the lack of order in the mucin-promoted motility observed for *P. aeruginosa* suggests that this form of surface motility might not depend on coordinated reversal of flagellar rotation. Interestingly, the bacterial cells from the edge of the mucin motility zones resembled cells in a biofilm, as bacteria lose their flagella and cells are piled on top of each other inside a growing biofilm. Moreover, various aspects, such as quorum sensing, shown to be important in the formation of the mucin motility colony also play important roles in biofilm formation (7, 38). We are currently testing normal swimming mutants defective in biofilm production as well as hyper-biofilm formers for altered surface motility on the mucin plates.

Previously, Overhage et al. performed microarray analyses of *P. aeruginosa* from the leading edge of a swarm compared to bacteria growing in identical medium under swimming conditions (11). Their study led them to identify major changes in gene expression patterns in swarming cells. In particular, they demonstrated that swarmer cells overexpressed a large number of virulence-related genes, including those encoding the T3SS and its effectors, those encoding extracellular proteases, and those associated with iron transport (11). Analysis by RT-qPCR of gene expression of *P. aeruginosa* taken from the mucin-mediated surface motility colony revealed that, in contrast to the results seen with swarming cells, genes encoding the T3SS and its effectors and those involved in phenazine biosynthesis were downregulated whereas genes encoding the T2SS and the exoproducts that this system secretes were upregulated. The important role of these virulence factors in *P. aeruginosa* pathogenesis has been demonstrated in various *in vivo* respiratory infection models (39–41). Significant upregulation of the *oprH*, *pmr*, and *arn* operons, which are not dysregulated in swarmer cells, was also observed. Activation of these operons is known to induce bacterial resistance to polymyxins and cationic antimicrobial peptides in response to low-Mg^2+^ conditions by controlling the addition of aminoarabinose to lipid A, thereby reducing the net negative charge of lipopolysaccharide (LPS). Consequently, the reduced net negative charge on the bacterial cell surface limits its interaction with and increases resistance to polycationic peptide antimicrobials (24, 42). The increased expression of virulence- and resistance-associated genes in these cells was not simply due to the presence of mucin, as no dysregulation of these genes was observed in *P. aeruginosa* grown in liquid MSCFM with or without 0.4% mucin (data not shown).

RT-qPCR revealed that *P. aeruginosa* taken from the middle of the mucin-promoted motility zones demonstrated downregulated expression of genes encoding pyoverdine and pyochelin biosynthesis, whereas cells at the edge of the motility zone upregulated expression of these genes (Table 2). This finding was unexpected, since the middle of the motility zone is green and the edge is white. We are currently unable to identify a mechanism for this observation; iron limitation, which would enhance the expression of pyoverdine/pyochelin genes, does not seem to provide an explanation, since the addition of (up to 640 µM) exogenous iron to the mucin-MSCFM plates did not alter the green pigmentation in the middle of the surface motility colony (data not shown).

Surface motility is complex and is dependent on the interplay of a variety of factors, including physical surface properties, the nutritional composition of the agar plates, bacterial cell signaling, and cell morphology. Based on these results, we suggest that mucin may be promoting a highly modified form of swarming or more likely a new form of surface motility. As there are so many distinguishing features of this form of surface motility, we suggest the name “surfing motility” based on the wetting properties of mucin and the appearance of the motility front as the motility zone progressed over time. Future studies may permit more in-depth understanding of the surface motility promoted by mucin in *P. aeruginosa*.

## MATERIALS AND METHODS

### Bacterial strains and growth conditions.

The *P. aeruginosa* strain PA14 wild type (WT) and the PA14 transposon mutants were obtained from Harvard University (43). The *P. aeruginosa* strain PAO1 WT and PAO1 transposon mutants were obtained from the University of Washington (44). Cultures were routinely grown in MSCFM (SCFM [18] without NH_4_Cl) or BM2 medium (62 mM potassium phosphate buffer [pH 7], 0.1% [wt/vol] Casamino Acids [CAA], 2 mM MgSO_4_, 10 µM FeSO_4_, 0.4% [wt/vol] glucose). Gentamicin at 15 µg/ml, kanamycin at 200 µg/ml, or tetracycline at 50 µg/ml was added to growth media for transposon maintenance.

### Swimming and swarming assays.

Swimming and swarming of *P. aeruginosa* WT and mutant strains were examined on MSCFM or BM2 plates containing 0.3% (wt/vol) agar (Difco) or 0.5% (wt/vol) agar, respectively, as previously described (11). As 0.1% (wt/vol) CAA was too low a concentration to support swarming of strain PAO1, a higher concentration of CAA (0.5% [wt/vol] [11]) was used to promote the ability of strain PAO1 to swarm.

### Mucin-promoted motility assays.

The surface motility of *P. aeruginosa* was examined on MSCFM or BM2 plates containing 0.2 to 0.3% (wt/vol) agar and 0.05 to 1.0% (wt/vol) sterilized porcine stomach mucin (Sigma). To sterilize mucin, dry powdered mucin was placed in a flask, covered with 95% ethyl alcohol, and heated at 70°C for 24 h (45). Sterile mucin was obtained by evaporating the alcohol. When necessary, herring sperm DNA (Sigma) (1.4 mg/ml) was also added to the mucin-containing MSCFM or mucin-containing BM2 plates (46). After mucin and DNA were added, the ingredients were stirred for approximately 15 min to help them dissolve. The plates were dried for 1 h and spotted with 1 µl of mid-logarithmic-phase cultures and incubated at 37°C for 13 to 15 h. The resultant colonies were analyzed by measuring the agar plate coverage. To test the effects of the carbon source, glucose was replaced by equimolar amounts of the carbon source of interest, with aspartate provided as the nitrogen source. To test the effects of the nitrogen source, total free amino acids were replaced with an equimolar amount of a single amino acid. Since the surface motility zones were easily measured at 0.4% (wt/vol) mucin and since we were able to obtain consistent results at this mucin concentration, we screened all flagellar, type IV pilus, rhamnolipid, quorum-sensing mutants by the use of this mucin concentration. We also used 0.4% (wt/vol) mucin for microscopic and genetic analyses. To measure the motility zones as a function of time, recording was initiated at the fifth hour, as this was approximately the time when motility colonies on all of the plates could be clearly observed and measured.

### Growth curves.

*P. aeruginosa* cells were grown overnight in MSCFM and diluted in fresh MSCFM with 0% to 0.8% (wt/vol) mucin. Flasks were shaken at 37°C, and aliquots were withdrawn every hour and serially plated onto LB agar plates. Alternatively, growth measurements using various carbon and nitrogen sources were performed by adding 5 µl of overnight cultures in MSCFM to 195 µl of fresh MSCFM with the appropriate carbon and nitrogen sources in 96-well microtiter plates. The growth of these cultures at 37°C under shaking conditions was monitored with a Tecan Spectrofluor Plus reader by determining the absorbance at 620 nm every 20 min for 14 h.

### Electron microscopy.

Carbon-coated grids were gently placed on the edge or center of the swarming or mucin-promoted surface motility colonies. After 2 min, the grids were removed and washed with distilled water and stained with 1% uranyl acetate. The negatively stained cells were visualized with a Hitachi H-7600 Transmission electron microscope (UBC Bioimaging Facility).

### Real-time quantitative PCR (RT-qPCR).

Total RNA from *P. aeruginosa* strain PA14 was harvested under the following conditions: (i) the leading edge of the surface motility colonies was maintained on MSCFM with 0.4% (wt/vol) mucin; (ii) cells swimming within the agar from MSCFM-swimming plates containing 0.3% (wt/vol) agar were incubated for 20 h at 37°C. Subsequently, RNA was isolated using RNeasy minicolumns (Qiagen) and treated with DNase I (Invitrogen) to remove contaminating genomic DNA. Three micrograms of total RNA was combined with 0.5 µM deoxynucleoside triphosphates (dNTPs), SUPERase-In (Ambion) (500 U/ml), and 10 µM dithiothreitol (DTT) in 1× reaction buffer and reverse transcribed with Superscript II reverse transcriptase (Invitrogen). The resultant cDNA was used as a template for qPCR. Analysis was carried out using an ABI Prism 7000 sequence detection system (Applied Biosystems) and a two-step RT-qPCR kit with SYBR green detection (Invitrogen). Fold change was determined using the comparative threshold cycle (*C_T_*) method by comparison to the *rpoD* housekeeping gene.

## SUPPLEMENTAL MATERIAL

Figure S1 Growth of *P. aeruginosa* strain PA14WT in liquid MSCFM with 0.4% mucin and various concentrations of DNA. Download Figure S1, TIF file, 0.1 MB

Figure S2 Growth of *P. aeruginosa* strain PA14 WT in liquid MSCFM with various concentrations of mucin. Download Figure S2, TIF file, 0.1 MB

Figure S3 Comparison of the levels of resistance of *P. aeruginosa* to polymyxin B determined on swimming and mucin-containing plates. Polymyxin B antibiotic (9 mg/ml) discs were placed in the middle of MSCFM plates with 0.3% agar and 0.4% mucin (A) and MSCFM plates with 0.3% agar only (swimming) (B). Mid-logarithmic-phase cultures of *P. aeruginosa* were spotted into the agar at 4 spots equidistant from the disc. The plates were incubated for 15 h at 37°C, and inhibition zones were determined. Inhibition zones were assessed as the distance from the closest bacterial growth front to the edge of the disc. The data demonstrate that swimming cells approach much more closely to the polymyxin disc than mucin-enhanced motility cells, indicating that the latter are likely more resistant to polymyxin. Download Figure S3, TIF file, 0.2 MB
