# Supplementary figures and images for: Mucin Promotes Rapid Surface Motility in Pseudomonas aeruginosa
Source: mBio. 2012 May 1;3(3):e00073-12. doi: 10.1128/mBio.00073-12 (PMC3569861; doi:10.1128/mBio.00073-12)

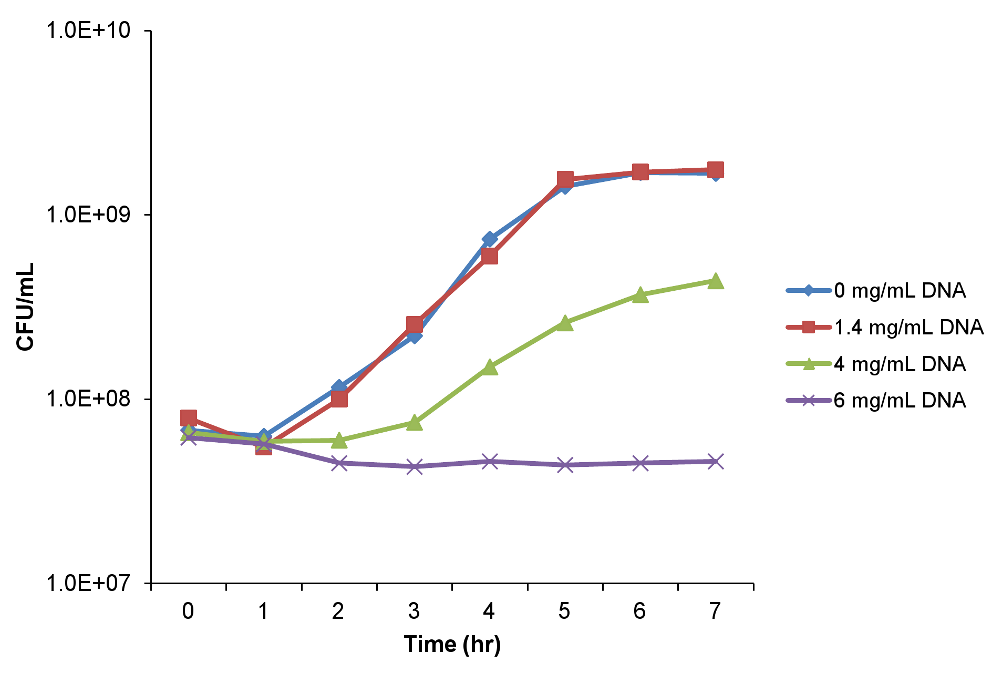

Supplement: Figure S1 — Growth of P. aeruginosa strain PA14WT in liquid MSCFM with 0.4% mucin and various concentrations of DNA. Download [file mbo002121270sf01.tif]

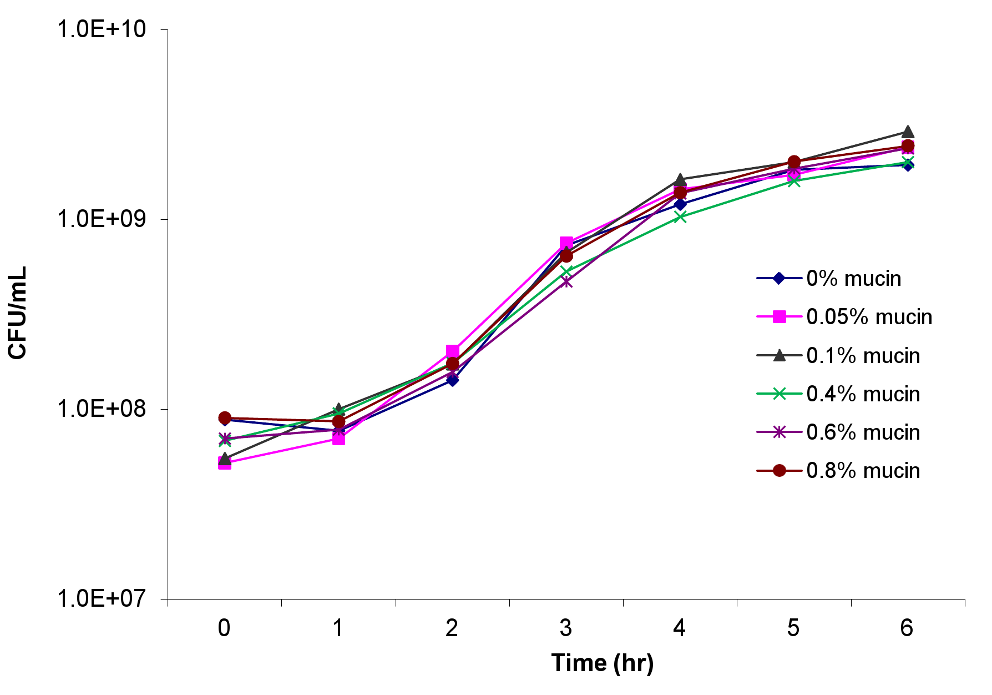

Supplement: Figure S2 — Growth of P. aeruginosa strain PA14 WT in liquid MSCFM with various concentrations of mucin. Download [file mbo002121270sf02.tif]

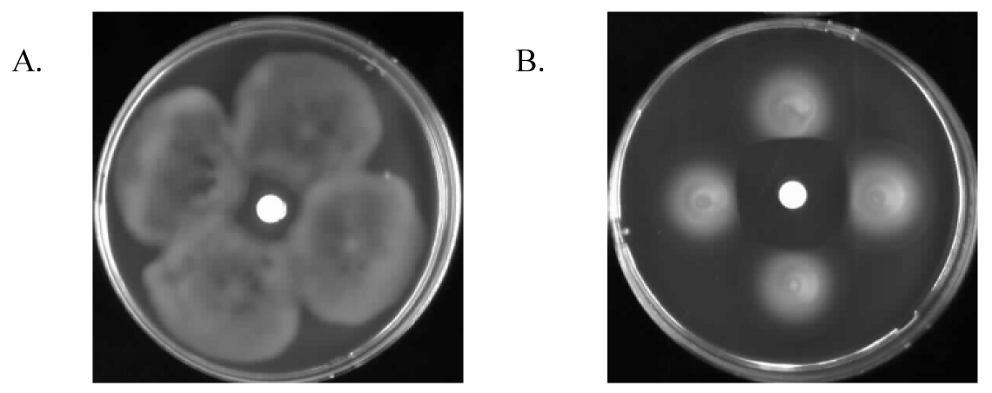

Supplement: Figure S3 — Comparison of the levels of resistance of P. aeruginosa to polymyxin B determined on swimming and mucin-containing plates. Polymyxin B antibiotic (9 mg/ml) discs were placed in the middle of MSCFM plates with 0.3% agar and 0.4% mucin (A) and MSCFM plates with 0.3% agar only (swimming) (B). Mid-logarithmic-phase cultures of P. aeruginosa were spotted into the agar at 4 spots equidistant from the disc. The plates were incubated for 15 h at 37°C, and inhibition zones were determined. Inhibition zones were assessed as the distance from the closest bacterial growth front to the edge of the disc. The data demonstrate that swimming cells approach much more closely to the polymyxin disc than mucin-enhanced motility cells, indicating that the latter are likely more resistant to polymyxin. Download [file mbo002121270sf03.tif]
